# Moiré Phase Mapping in Twisted Bilayer Liquid Crystals with Reconfigurable Light Polarization States

**DOI:** 10.1002/adma.74825

**Published:** 2026-08-27

**Authors:** Jordan A. Wilson, Rui Huang, Allen Sun, Zixian Fang, Doreen Mwangombe, Keerthna Naina Rawat, Oliver Jacobs, Zhiwei Li

**Affiliations:** ^1^ Department of Chemistry and Biochemistry University of Maryland College Park Maryland USA

**Keywords:** dichroism, liquid crystals, moiré, polarization, twisted bilayers

## Abstract

Liquid crystals are crystalline materials with only orientational orders without long‐range positional orders, which have been broadly used in optical devices due to their capability in modulating light polarization. In developing practical optical devices, photolithography is widely used in patterning liquid crystals for sensing, anticounterfeiting, displays, and camouflage. These structures are fixed once made and creating dynamic multi‐domain optical materials is challenging. Here, we report the development of dynamic moiré patterns by vertical stacking assembly of two liquid crystals having designer domains and optical properties. Magnetic Fe_3_O_4_ nanorods were used as building blocks and assembled into liquid crystals under a magnetic field, which is driven by the preferential alignment of nanorods’ long axes to the field. Stacking two aligned nanorod films with controlled twist angles forms twisted bilayers that generate moiré superstructures in polarization space, producing angle‐dependent trajectories on the Poincaré sphere. This work demonstrates spatially resolved Stokes parameters and twist‐dependent photonic phases based on pixel‐level mapping of light polarization states. These moiré‐symmetry, twisted bilayer liquid crystals provide a route to active optical devices for dynamic photonic modulation, polarization filtering, and polarization‐encoded anticounterfeiting.

## Graphical abstract

Twisted bilayer liquid crystals are developed by vertical stacking assembly of two liquid crystals having designer domains and optical properties. These moiré‐symmetry, twisted liquid crystals demonstrate reconfigurable polarization states, moiré patterns and phases controlled by twist angles, providing a route to design active optical devices for dynamic photonic modulation, polarization filtering, and polarization‐encoded anticounterfeiting.

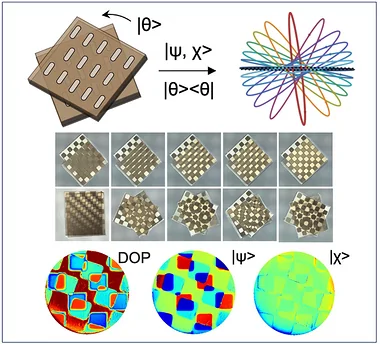

## 1 Introduction

Developing responsive optical nanomaterials present a powerful means to dynamically control the optical properties and symmetry of nanostructured materials. Anisotropic magnetic nanoparticles, such as nanorods and nanowires, can align under external magnetic fields, enabling field‐directed assembly and tunable optical anisotropy [1, 2]. These structural properties have been applied to develop optical devices, adaptive optics, and stimuli‐responsive materials. For example, previous studies have shown that magnetic nanorods can form liquid‐crystalline phases under external magnetic fields, producing macroscopic optical birefringence and polarization‐dependent transmission due to particle orientation [3, 4, 5]. Paramagnetic inorganic nanoparticles such as iron oxide nanorods are particularly attractive because their optical properties can be instantly and reversibly controlled by manipulating their orientation with relatively weak external magnetic fields. Due to the ability to remotely control optical effects and rapidly reconfigure particle alignment, magnetically responsive optical nanomaterials offer a distinct advantage in tuning symmetry and optical properties over photonic structures made by lithography or fabrication methods [6, 7].

Aligned rod‐like particles induce anisotropic scattering and absorption that alters the amplitude and polarization states of transmitted light, characterized via Stokes parameters [8, 9, 10]. Prior work has shown that polarization‐selective media composed of aligned nanorods responds strongly to particle geometry, concentration, and orientational order [11, 12], but most studies have focused on single‐layer systems with fixed optical properties once fabricated. Twisted bilayer structures add an extra degree of freedom for controlling electronic and optical properties. The term “twisted” is used to denote the twist‐induced misalignment and the resulting tunable out‐of‐plane symmetry between two independent layers. In anisotropic optical media, layer rotation has been predicted to spatially vary the optical axes and birefringence, producing chiroptical responses that depend on twist angle [13, 14]. Among these emerging materials platforms, moiré photonic lattices have gained great attention for their simple symmetry tuning by twisting two or more periodic lattices to engineer new photonic band structures and light‐matter interactions, which are not easily attainable in conventional Bravais‐lattice photonic lattices or metasurfaces [15, 16, 17]. Due to twist‐induced symmetry reconstruction, moiré photonic lattices have developed as a platform for a range of novel optical phenomena, including light localization, bound states in the continuum, and topological defects. One prominent property of the twisted photonic crystals is the moiré flat band formation at a “magic angle,” enabling slow‐light propagation over extremely narrow bandwidths [18]. Similarly, moiré bound states in the continuum (BICs) have been theoretically proposed in one‐dimensional photonic crystals, where flat‐band formation supports high‐Q resonance [19]. Recently, Wang et al. developed twisted nematic liquid crystals by rotating two periodically patterned surfaces and demonstrated the presence of highly tunable topological defects in nematic liquid crystals due to the moiré effects, leading to disclination lines and loops [20].

Despite this progress, a systematic understanding of how twist angle governs photonic phases and polarization states in twisted superlattices is still lacking, limiting the design of responsive active optical materials. Here, we report twisted bilayer liquid crystals (TBLCs) by stacking two layers of liquid crystals along a designer twist angle, in which the twist angle between two magnetically aligned liquid crystal films serves as a single, continuously tunable parameter for reconfiguring light polarization states. Specifically, we refer to our system as TBLCs, by analogy with twisted bilayer structures in 2D van der Waals nanomaterials, such as graphene. Under external magnetic fields, Fe_3_O_4_ nanorods align to form liquid‐crystalline assemblies that modulate light transmission and polarization. Using these assemblies as sublattices, we developed TBLCs superlattices in which varying the twist angle alone produces both complex moiré patterns and reversible polarization state tuning. Polarization measurements combined with Stokes parameter analysis, reveal a continuous evolution of polarization states, which can be visualized on a Poincaré sphere. Tunable polarization domains are further fabricated by integrating magnetic alignment with photolithographic patterning, enabling continuous, complex photonic phase transitions driven by structural rotation, reaching a level of reconfigurability that is difficult to achieve in conventional superstructures made by assembly or lithography alone. Our approach builds moiré liquid crystals from magnetically aligned Fe_3_O_4_, shifting the resulting optical response from a defect engineering toward continuous, reversible polarization state control. These twisted optical superstructures offer a versatile approach for designing tunable photonic devices, including optical modulators, polarization filters, and anti‐counterfeiting devices.

## 2 Results and Discussions

The vertical stacking assembly of liquid crystals made of aligned magnetic nanorods is illustrated in Figure 1a. Two liquid crystals served as sublattices and were stacked into bilayers along a twist angle, which provides a reconfigurable approach to actively tuning light polarization states. To characterize the nanorod morphology and sizes, we acquired representative transmission electron microscopy (TEM) and scanning electron microscopy (SEM) images of FeOOH and Fe_3_O_4_@SiO_2_ nanorods (Figures 1b and S1). Those nanorods were coated with a layer of silica through a sol–gel process and then reduced to Fe_3_O_4_@SiO_2_ by tetraethylene glycol at 260°C [21, 22]. As shown in Figure 1c, the product maintains a well‐defined rod‐like morphology. The surface silica layer plays an important role in stabilizing the colloidal dispersion of magnetic nanorods: it acts as a physical barrier to separate the magnetic cores, attenuates their magnetic dipole–dipole interactions, and prevents aggregation [23, 24, 25]. Magnetic nanorods are easily dispersed in various polar solvents, including water and alcohol, due to the abundant hydroxyl groups on the silica surface [26, 27]. Upon the application of an external magnetic field, the magnetic nanorods align themselves along the field direction, producing the rotational order needed for the formation of liquid crystals [28, 29]. To directly observe the alignment of the Fe_3_O_4_@SiO_2_ nanorods, they were dispersed in a UV‐curable precursor solution. Under an external magnetic field, the dispersion was exposed to UV light to initiate polymerization. To illustrate the liquid crystal configuration in the twisted bilayer, we performed confocal imaging by changing the focal plane between the twist interfaces (Figure 1d). The magnetic nanorods have unidirectional alignment in the two layers and form a twisted configuration between the interfaces, which was evidenced by the right image and the inset FFT image of superimposed layers in Figure 1d. The orientational uniformity of the magnetic nanorods was further quantitatively characterized using polarized UV–vis spectroscopy to extract the dichroic order parameter *S*, as detailed in Figure S2. The increase in the order parameter from shorter to longer wavelengths arises because the longer‐wavelength absorption is dominated by longitudinal transitions polarized along the nanorod long axis, which produce a much larger dichroic ratio *R* = *A*_∥_/*A*_⊥_ and thus a higher extracted *S*. At shorter wavelengths, this longitudinal contribution is mixed with transverse and other less‐directional transitions, so overlapping perpendicular absorption channels dilute the polarization contrast and artificially suppress the apparent *S*. Therefore, the low *S* value at short wavelength is mainly induced by isotropic absorption of the less‐directional transitions rather than the structure disorders. On the other hand, the large *S* value at long wavelength mainly measures the structure orders due to the well‐defined anisotropic longitudinal transitions, underpinning the good orientational uniformity of magnetic nanorods in the liquid crystal films. Figure 1e illustrates our experimental setup for measuring transmission spectra as a function of the twist angle between two vertically aligned films. We observed consistent decreases of transmission of nonpolarized light as the twist increased to 90° (Figure 1f). To analyze the dependence of transmittance on twist angle (*θ*), we normalized the intensity of transmitted light to the minimum and maximum values and plotted normalized intensity against the angle (Figure S3a). We observed a periodic change of the light intensity as the twist angle increases from 0° to 180°, which is proportional to cos^2^ *θ*. To evaluate the accuracy of twist angle in our experiments, we estimated the actual twist angle based on the measured light intensity changes. As shown in Figure S3b, a linear dependence was observed, and small errors in each measurement suggest a good accuracy and reproducibility of the twist angle control in reconfiguring the polarization states. Figure S4a illustrates unpolarized light passing through a linear polarizer and then a magnetically aligned film. The dependence of transmittance on the rotation angle is shown in Figure S4b and the corresponding images are given in Figure S4c. Comparing the intensity of transmitted light in Figures 1f and S4b, we observed a consistent relationship between the intensity and the twist angle, both of which suggest the intensity being proportional to cos^2^ *θ*. This agreement implies that the liquid crystal films containing aligned nanorods serves as a polarizer that convert non‐polarized light into polarized light. To understand the dependence, we introduce the Bra‐ket notation (see Methods) to derive an analytical solution for predicting the transmittance in the TBLCs. Consider the polarization effect of single‐layer liquid crystals and imagine the polarization direction of the transmitted light being perpendicular to nanorod alignment. The polarization effect can be described as a polarization operator *P*(θ) =  |θ〉〈θ|, where *θ* is orientation angle of the nanorods in the single‐layer liquid crystals or the twist angle in the TBLCs. For non‐polarized light propagating along *y*‐axis, the first layer has nanorods aligned along *z*‐axis and the second layer is twisted along the angle *θ*. The associated polarization operator can be expressed as |*x*〉〈*x*| and |θ〉〈θ|, respectively. After applying the two operators on the non‐polarized light, the polarization state of the transmitted light through the TBLCs at a twist angle of *θ* can be defined by cosθ|θ〉 and the transmittance can be predicted by the expectation value (cos^2^ *θ*) of the polarization state. This numerical solution derived from the Bra‐ket notation suggest that the light intensity of non‐polarized light after interacting with the TBLCs at a twist angle *θ* can be predicted by cos^2^ *θ*, which is consistent with our experimental measurements in Figure S3.

**FIGURE 1:**
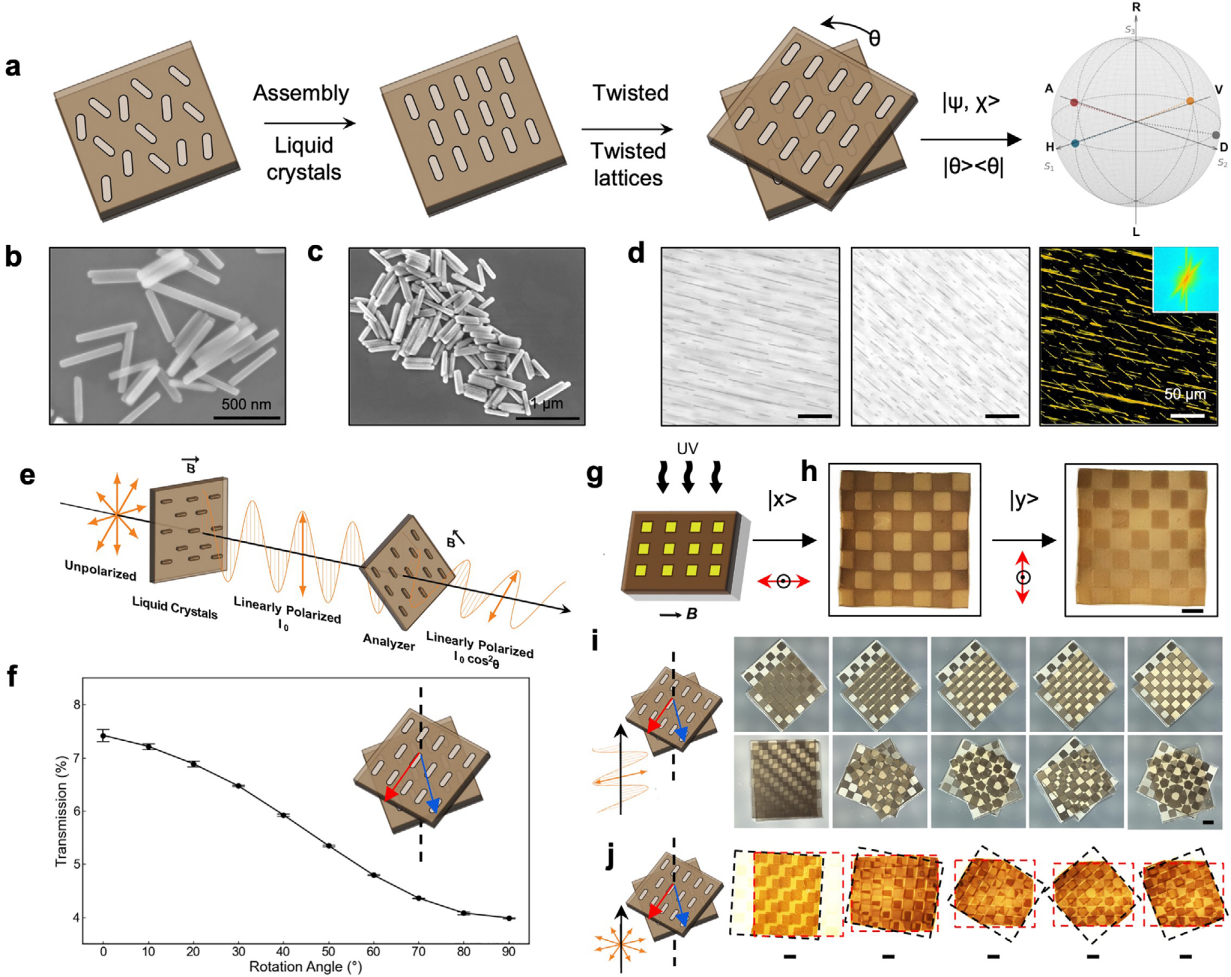
Twisted liquid crystals. (a) Schematic illustration of a twisted moiré lattice altering light polarization states. SEM images of (b) FeOOH nanorods and (c) Fe_3_O_4_@SiO_2_ nanorods. Confocal images of the Fe_3_O_4_@SiO_2_ nanorods aligned into liquid‐crystal phases embedded within a polymer matrix of first (left panel) and second twisted (middle panel) and superimposed layers (right panel), showing the rotational order of magnetic nanorods. Inset: FFT image of superimposed layers, demonstrating the twisting order of the two layers. (e) Schematic illustrating non‐polarized light transmitted through the twisted liquid crystal films at different twist angles. (f) The corresponding transmission spectra as a function of the twist angle of the twisted liquid crystal films. (g) Schematic illustration of the preparation of liquid crystal films using photolithography. (h) Digital image of the liquid crystal film under *x*‐ and *y*‐polarized light. Digital images of the twisted films at different twist angles under (i) polarized light and (j) non‐polarized light illumination. Scale bars: 2 mm.

By following a photolithography method as illustrated in Figure 1g, we prepared two identical liquid‐crystal films that feature square domains with orthogonally aligned nanorods in the two different types of domains. Under polarized light in Figure 1h, the domain with different contrasts contains the orthogonally aligned magnetic nanorods and switching light polarization leads to contrast changes due to polarization effects of the liquid crystal films. TBLCs films show complicated moiré patterns that can be tuned between different twist angles under linearly polarized light (Figure 1i). More interestingly, even under nonpolarized light, we demonstrated complicated moiré patterns and consistent transformations between these moiré patterns by changing twist angles (Figure 1j).

To study the twist angle‐dependent transmission properties in the TBLCs, we investigated light polarization states of one single liquid crystal film using a rotating quarter‐wave plate method (Figure 2a) [30]. The experiment was conducted at 535 nm to evaluate the influence of nanorod orientation on the degree of polarization (DOP). Magnetic nanorods of three different aspect ratios were synthesized, and the sample with an average length of 410 nm and a width of 140 nm exhibited the highest DOP, as shown in Figure 2b. Transmittance spectra of the liquid crystal films made of nanorods with different aspect ratios were measured under both linear polarizer and unpolarized light (Figure S5a,b). Spatial maps of retardance, birefringence (Δ*n*), and diattenuation were acquired for single‐layer liquid crystals with varying nanorod aspect ratios and concentrations to quantify their optical properties. The measured linear retardance was equal to the total retardance, indicating that the TBLCs behave as purely linear retarder. Consequently, the circular retardance is effectively zero (Figures S6 and S7). The derived the retardance and diattenuation based on Mueller matrix method are shown in Tables S1–S4. The effective birefringence decreases as concentration increases, even though the system remains strongly dichroic. Such decrease is mainly attributed to the strong polarization‐selective extinction and high diattenuation at high nanorod concentrations as well as possible concentration‐induced bundling in the liquid crystal films. In an absorptive liquid crystal film with large Δ*n*, the sample no longer acts as a purely dichroic polarizer but also as a waveplate that continuously rotates the polarization state as light propagates, converting incident linear polarization into elliptical polarization. As a result, the transmitted light is less purely linearly polarized for samples with high birefringence, and the measured polarization decreases at low concentrations. On the other hand, both DOP and diattenuation exhibit an approximately linear increase with increasing nanorod concentration (Tables S3 and S4), reflecting the growing and dominating contribution of anisotropic absorption and dichroism in the polarization effect in the system. Interestingly, we conclude that the polarization of transmitted light through the liquid crystals is observed to be perpendicular to the nanorods’ alignment. Due to anisotropic liquid crystal's dichroism, the diattenuation of liquid crystals depends on light's polarization and propagation direction, leading to the polarization of transmitted light being perpendicular to rod alignment [31, 32]. Using a wire grid polarizer as an example, when an electromagnetic wave encounters parallel wires, the electric field component parallel to the wires drives conduction electrons along the wires, suppressing the transmission of that component through absorption and reradiation [33, 34, 35]. In comparison, the perpendicular field component does not efficiently drive electron motion and therefore passes through with relatively little to no attenuation. In magnetic nanorod systems, the liquid crystal phases induce birefringence and dichroism of the solid films [36], resulting in the transmitted light to be polarized perpendicular to the nanorods’ alignment direction. As a result, our wavelength‐dependent measurements revealed pronounced DOP enhancement across the visible spectrum (Figures 2c and S8). The correlation between nanorod alignment and light polarization can be represented using a Poincaré sphere (Figure 2d), demonstrating a consistent transition of polarization states. Concentration‐dependent measurements showed that DOP increased with nanoparticle concentration ranging from 0.82 mg/mL to 6.56 mg/mL (Figures 2e–g and S9a,b). Figure 2h demonstrates that the DOP increases at three distinct wavelengths (red, green, and blue) as the Fe_3_O_4_ nanoparticle concentration rises.

**FIGURE 2:**
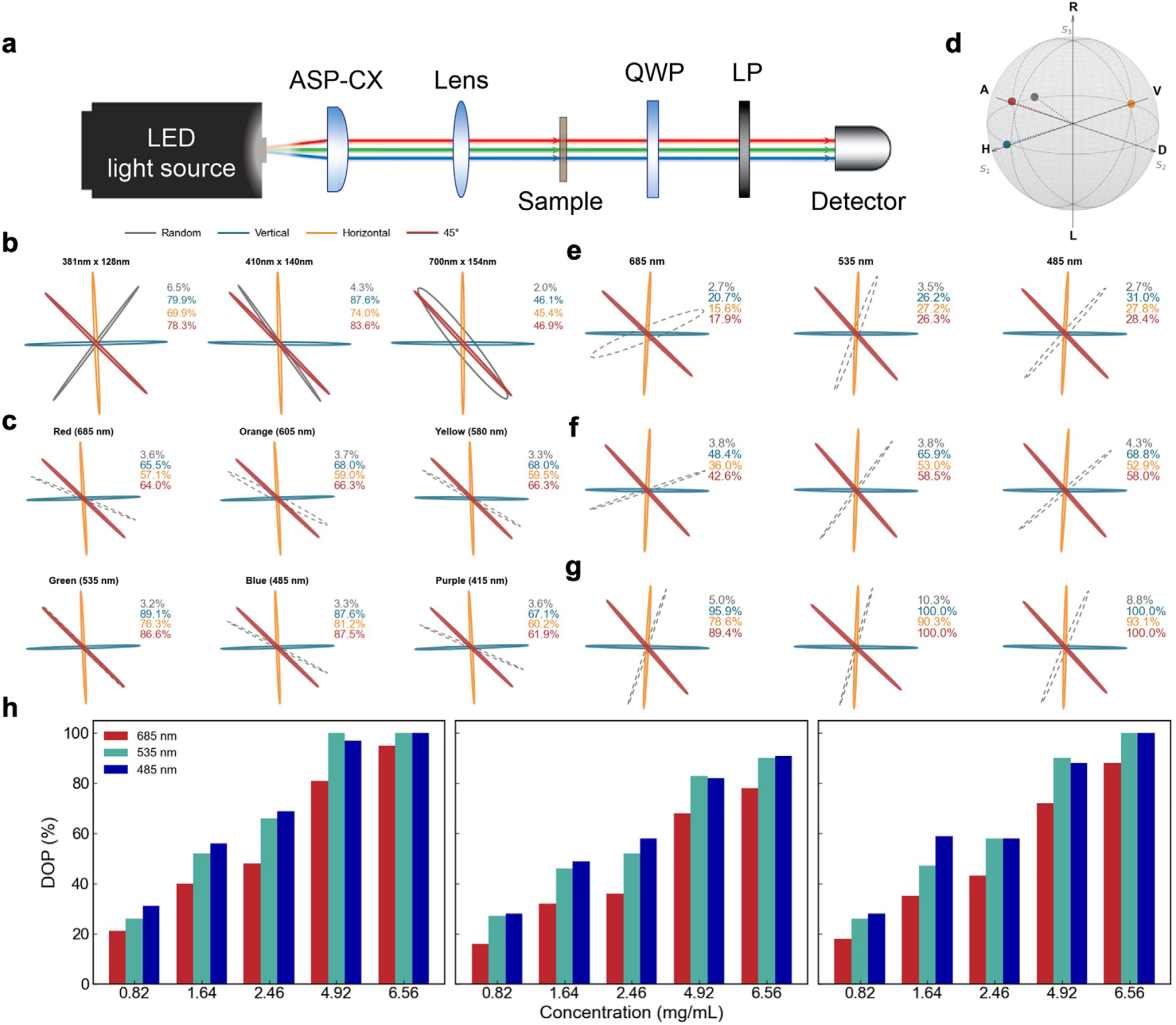
Polarization analysis of the liquid crystals. (a) Experimental setup of the quarter‐wave plate method used to study light polarization states. (b) Polarization ellipse measurements for three samples with different sizes: 381 nm × 128 nm, 410 nm × 140 nm, and 700 nm × 154 nm under excitation of light at 535 nm. (c) Degree of polarization (DOP) of nanorods (410 nm × 140 nm) measured under illumination with six visible wavelengths. (d) The corresponding Poincaré sphere of the nanorods. (e–g) Polarization ellipse measurements of 410 nm × 140 nm nanorods at varying Fe_3_O_4_@SiO_2_ nanoparticle concentrations: (e) 0.82 mg mL^−1^, (f) 2.46 mg mL^−1^, and (g) 6.56 mg mL^−1^ at 535 nm. (h) The dependence of DOP of the nanorods (410 nm × 140 nm) on Fe_3_O_4_ nanoparticle concentrations under vertical, horizontal, and diagonal alignment from left to right.

Building on the established polarization behavior of Fe_3_O_4_ nanorods, spectroscopic and DOP analyses were conducted on TBLCs formed by stacking bilayer assemblies. Twisted bilayer configurations introduce periodic optical interference that resembles moiré superstructures commonly observed in electronic systems [37, 38]. Four stacking configurations were examined with AA and BB referring to inversion symmetry, and AB and BA being inversion symmetry broken (Figure 3a). Films denoted with A have a concentration of 1.64 mg/mL, and films denoted with B have a higher concentration of 4.92 mg/mL. For each configuration, polarization ellipse measurements were obtained by incrementally rotating the first layer from 0° to 180°, while the second layer remained fixed at 0°. The polarization ellipse results are shown in Figure 3b. The inversion‐symmetric BB configurations exhibited a slightly higher DOP and azimuth compared to the AA configuration. Notably, when inversion symmetry was broken, the AB configuration showed minimal change in the azimuth of the polarized light. In contrast, the BA configuration showed a clear full rotation of polarized light. DOP and azimuth exhibit reproducible angular dependence as the rotation progresses from 0° to 180° (Figure 3c). The ellipticity (*χ*) is plotted as a function of the twist angles for both inversion‐symmetric and inversion symmetry‐broken configurations, together with DOP and azimuth (Figure S10a,b). The inversion‐symmetric configurations showed a wider range of DOP at the rotation angles, whereas the DOP for the inversion symmetry breaking configuration decreased from 0° to 180°. It is also clear to see the drastic change in azimuth when switching from the AB to the BA configuration, demonstrating that the polarization ellipse changes based on the configuration when stacking two liquid crystal films of different concentrations and optical properties. From Stokes parameter analysis, we observed that when the rotation exceeded 90°, the handedness of the ellipse changed from left‐ to right‐handed polarization in all configurations. This is the result of the combination of vertically and horizontally polarized light, which generates ellipticity. Only when all configurations are fixed at 0° or 180° will we have completely linearly polarized light. Figure 3d displays the trajectory on the Poincaré sphere, revealing the continuous evolution of polarization orientation across all stacking configurations and confirming coherent polarization transfer between aligned nanorod layers.

**FIGURE 3:**
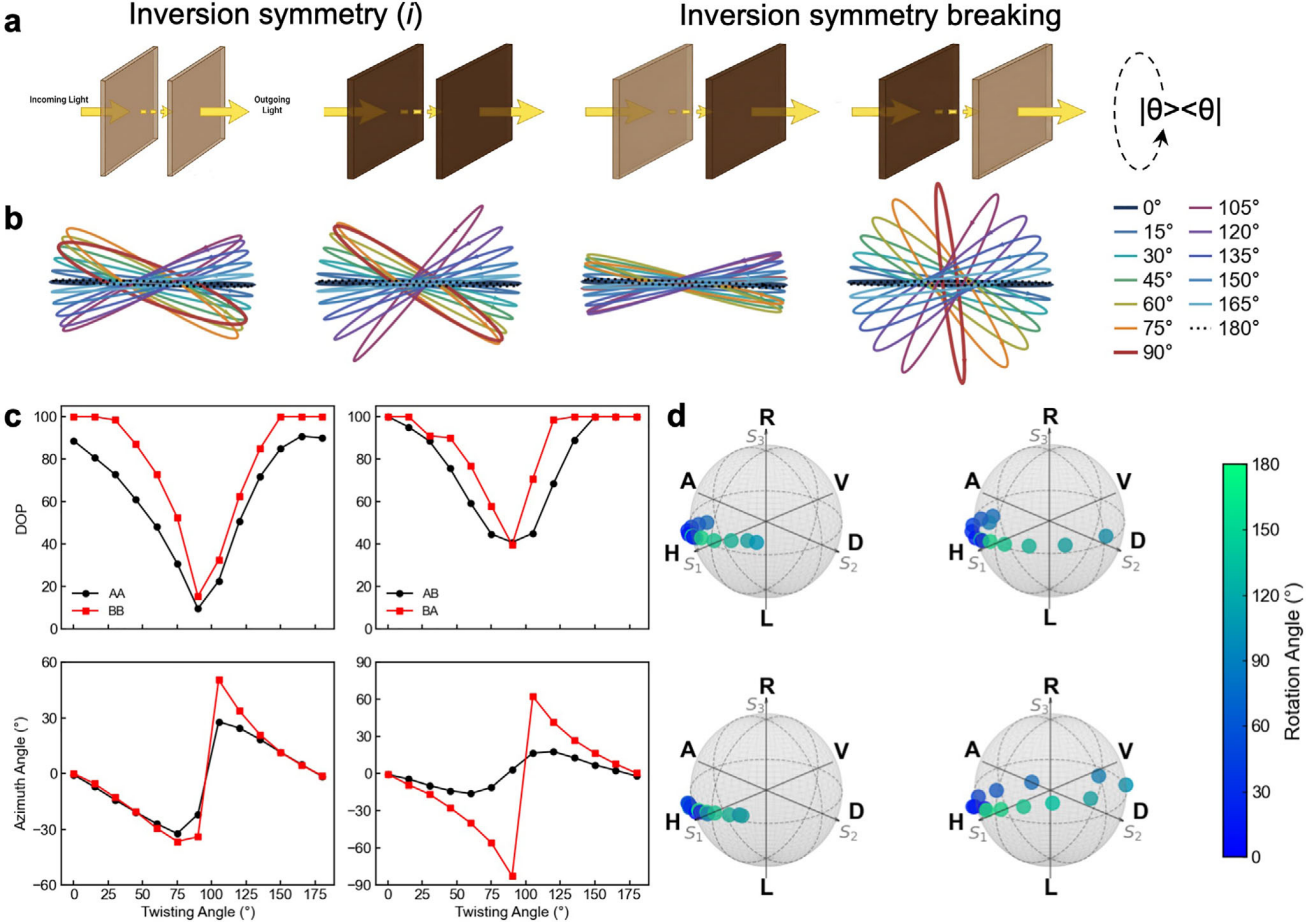
Spectroscopy and degree of polarization (DOP) studies of twisted liquid crystals in twisted bilayers. (a) Schematic illustration of bilayer stacking of liquid crystal films made of low (light film A) and high (dark film B) nanorod concentrations. (b) The corresponding polarization ellipse measurement by changing the twist angle from 0° to 180°. (c) DOP and polarization orientation plots for all stacking configurations. (d) The Poincaré sphere illustration of AA and BB (top) and the AB and BA (bottom) configurations.

Inspired by the twist angle‐dependent optical properties, we developed a reliable approach to patterned moiré liquid crystal materials using lithographic techniques. As schematically shown in Figure 4a, a solution containing magnetic nanorods and polymer precursor was first sandwiched between a glass coverslip and a glass slide [39]. A photomask was then placed on top of the sample, followed by the application of a magnetic field. Upon exposure to UV light, the nanorods in the uncovered regions were oriented along a specific direction within the film plane [40]. The glass slide was flipped and rotated 90° (in‐plane), then re‐exposed to UV light in the presence of a magnetic field. In this way, we prepared thin films with magnetic nanorods orthogonally aligned in two different types of domains that showed different transmittances for polarized light. Figure 4b displays images of two liquid crystal films with square (top two panels) and circle patterns (bottom two panels) under polarized light illumination. The areas cured during the first exposure appear dark under linearly polarized light, owing to the parallel arrangement of the nanorods relative to the transmission axis of the polarizer. Changing the light polarization from vertical to horizontal direction switched the contrast in the two domains. The twisted configuration also allows us to read a polarization‐coded photonic pattern as demonstrated in Figure S11. A plain liquid crystal with unidirectional nanorod alignment was stacked on a patterned liquid crystal (Figure S11a). Under non‐polarized light, the photonic pattern can be barely observed although the nanorods were orthogonally aligned in different domains (Figure S11b). By stacking the plain liquid crystal on the top of the patterned film, contrast between the underlying domains becomes apparent so that the coded pattern is readable. Its contrast also evolves continuously as the top film is twisted relative to the bottom patterned film (Figure S11c–e). In Figure 4c, we delineated the formation of twisted lattices by stacking two identical patterned liquid crystal films. Experimental observations of sliding and rotating lattices were shown in Figure 4d,e. Stacking two identical liquid crystal lattices produces larger‐scale moiré patterns that repeat periodically across the plane under the illumination of non‐polarized light. When extended over a sufficiently large area, these structures exhibit two‐dimensional symmetries known as wallpaper groups, which classify periodic patterns based on the symmetry operations they contain, including translations, rotations, reflections, and glide reflections [41]. In total, there are 17 possible plane symmetry groups that describe all repeating patterns in two dimensions. In this work, the moiré patterns were analyzed to identify their symmetry operations and assign the corresponding group classifications. Using the Hermann–Mauguin notation, the symmetry groups begin with *p* or *c*, indicating a primitive or centered lattice, followed by a number *n* specifying the order of rotational symmetry, and additional symbols such as *m*, *g*, or *1*, which denote mirror symmetry, glide reflection symmetry, or the absence of these reflection symmetries [42]. The sliding and rotation of these patterned films reveal a relationship between contrast and degree of polarization, as well as between twist angle and symmetry group. In the case of the square moiré lattice, rotation from 0° to 90° results in a fluctuating change in contrast within the moiré pattern. When the first film is rotated over the fixed second film, regions that appear lighter indicate parallel alignment of nanorods in both films and a low DOP. Conversely, darker regions indicate nanorods' perpendicular alignment in the twisted films, which is associated with a higher DOP. Regarding symmetry groups, only specific twist angles produce different symmetry groups although various shapes and patterns es rotation. As an example, by arranging the placement of the dots in the circle lattices, it changes the symmetry of the moiré patterns.

**FIGURE 4:**
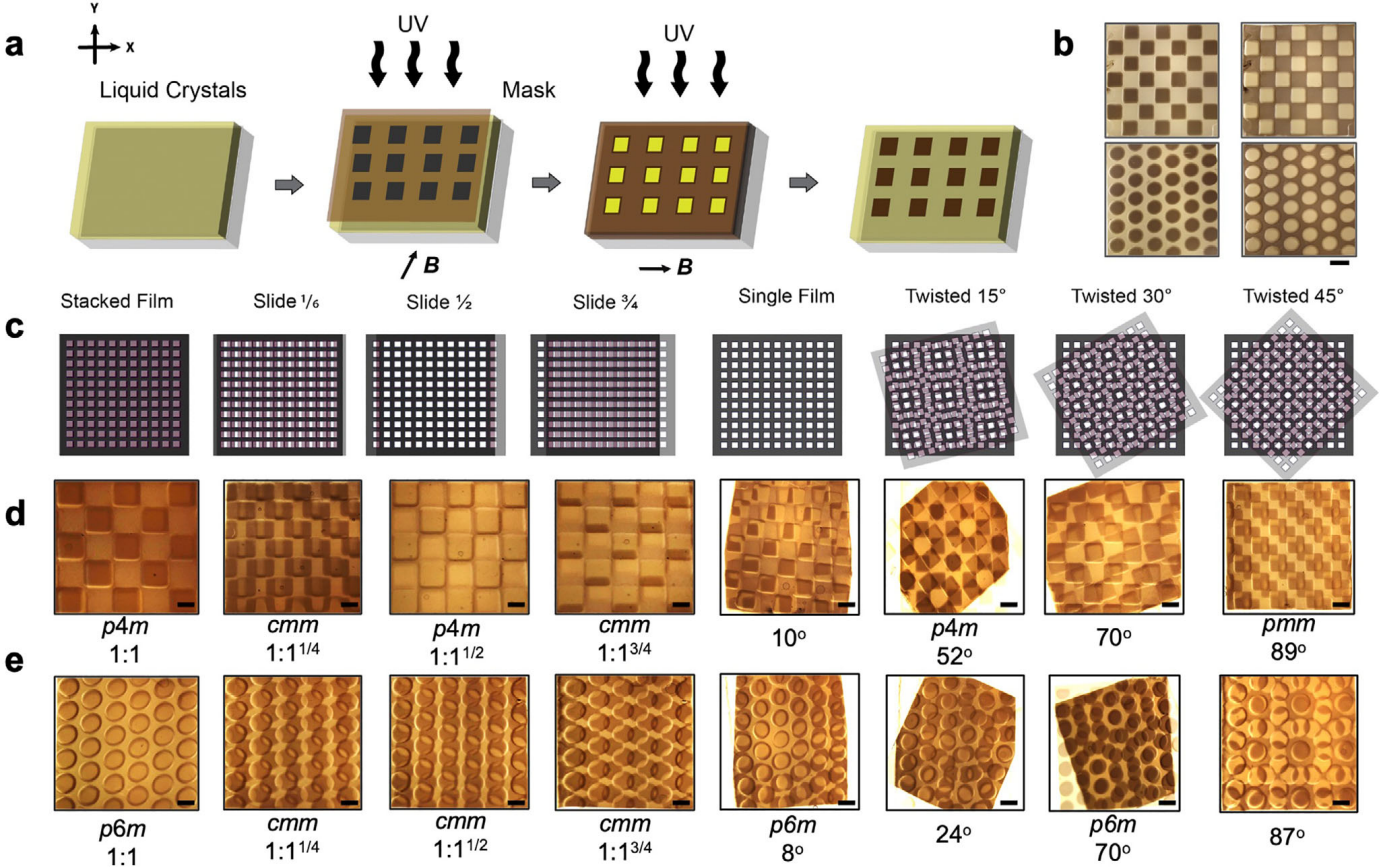
Fabrication of twisted liquid crystal devices. (a) Schematic illustration of the lithographic process used to fabricate liquid crystal films with spatially varying orientational order. (b) Polarized optical images of square and circular patterns exhibiting contrast switching upon changing light polarization from vertical (left two images) to horizontal (right two images) directions. (c) Schematic representations of moiré lattice formation. Left: Stacked moiré lattices generated by lateral sliding of one layer relative to the other by 1/6, 1/2, and 3/4 of the lattice period (from left to right). Right: A single‐layer thin film and stacked moiré lattices with twist angles of 15°, 30°, and 45° from left to right. (d) Moiré patterns formed by sliding and rotating square lattices with their symmetry groups. (e) Moiré patterns formed by sliding and rotating circular patterns with their approximate symmetry groups. Some incommensurate moiré patterns retain rotational order but lack exact translational periodicity. Scale bars: 2 mm.

To explain how a simple structural twist leads to the complex characterized LCs, we developed a pixel‐based algorithm to derive light polarization states of each pixel and to map the twist angle‐dependent moiré domains and phases. We took images of the TBLCs using the optical setup shown in Figure 2a by rotating the quarter wave plate from 0° to 180°. To investigate moiré pattern formation in TBLCs, we prepare moiré patterns that exhibit consistent contrast changes under distinct twist angles (Figures 5a,b and S12–S14). Domain boundaries between regions of differing orientation in single‐layer liquid crystal film were characterized by optical microscopy, revealing a sharp transition from vertical to horizontal alignment through a narrow intermediate region. We found the transition width to be ∼4 µm (Figure S15). Based on these images, we derived the pixel intensity and analyzed the light polarization states of each pixel. We studied the polarization of magnetically aligned liquid crystals by measuring the polarization of different domains in moiré patterns, as shown in Figure 5a (15°) and Figure 5b (45°). These images revealed four distinct contrast domains within the square stacked moiré lattices, as shown in Figure 5c. In Figure 5d, pixel‐resolved intensity analysis was performed by normalizing the pixel intensity of that specific domain using (*I*_θ_ − *I*_min_)/(*I*_max_ − *I*_min_), where *I*_θ_ is the intensity of the pixel at the corresponding waveplate rotation angle (*θ*). The image and intensity‐*θ* plot for a twist angle of 45° were shown in Figure 5e,f, respectively. Comparing the phase and images in different twist angles suggested a twist‐dependent optical phase for TBLCs made of two identical films. Based on periodic curve fitting in Figure 5d,f, we were able to derive important parameters of light polarization states, including DOP, polarization direction, and ellipticity of transmitted light in different domains. One example of the Poincaré sphere and reconstructed polarization ellipse of the four domains in Figure 5c are shown in Figure 5g,h, respectively. From these measurements, we reconstructed spatial maps of DOP, polarization azimuth ψ, and ellipticity angle χ for 15°, 30°, and 45° as shown in Figures 5i,j and S16. These results demonstrate the feasibility of spatial polarization mapping in aligned Fe_3_O_4_@SiO_2_ nanorod films. The DOP mapping in Figures 5i,j, and S16a demonstrated well‐defined domains of different degrees of polarization, and such twist‐dependent spatial distribution of polarization degrees explains the formation of moiré patterns in Figure 4d,e. Taking the twist angle of 30° as example, we were able to analyze their normalized intensity as a function of the quarter‐wave plate angle *θ* (Figure S16b,c) and constructed the corresponding Stokes vectors on the Poincaré sphere (Figure S16d) and the associated polarization ellipses from the measured Stokes parameters (Figure S16e). At the twist angle of 45°, we observed less DOP variations in the twisted films largely because the nanorods in the two domains are misaligned at 45° in the twisted films. However, in the ψ‐mapping in the middle panels of Figure 5i,j, the dramatic changes in polarization azimuth across −90° to 90° suggest detailed structures of the nanorods’ alignment in the twisted films (Figure S17a,b). More specifically, while DOP mapping produces relative twist angles between nanorods in overlapped domains, the ψ‐mapping delivers more detailed information on the spatial alignment and twist directions of nanorods. In contrast, χ‐mapping is trivial in different twist angles as demonstrated in the right panels in Figure 5i,j, suggesting that the twisted superstructures mainly induce linearly polarized light with relatively small ellipticity.

**FIGURE 5:**
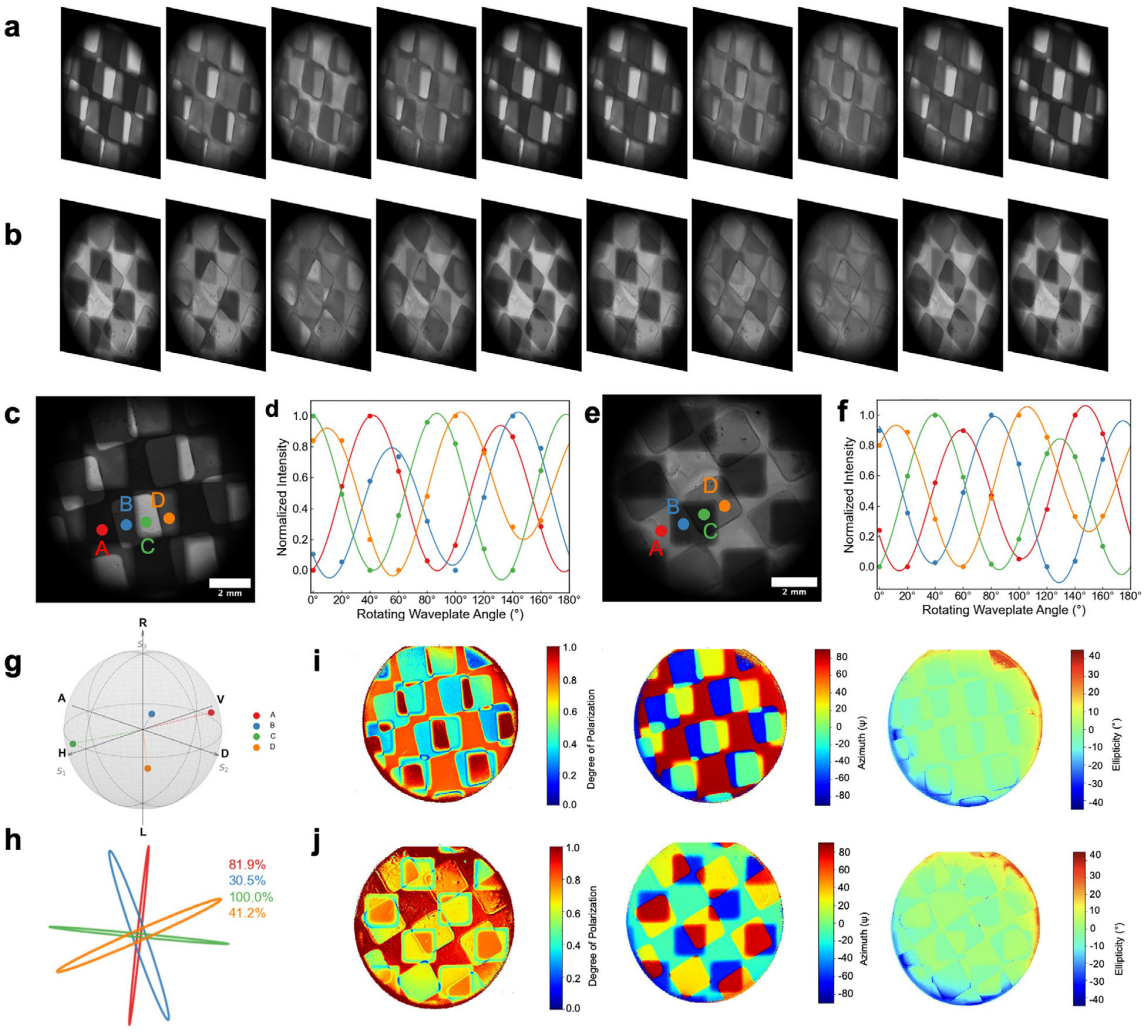
Moiré phase mapping. (a and b) Grayscale optical images of stacked moiré lattices with relative twist angles of (a) 15° and (b) 45°. (c) Four domains in the 15° twisted lattice and (d) their normalized intensity plot. (e) Domains and (f) angular intensity for the 45° twisted lattice. The intensity modulation by quarter‐wave plate rotation enables extraction of the polarization state. (g) Poincaré sphere representation of the Stokes vectors for four domains in the 15° twisted lattice. (h) Reconstructed polarization ellipses from the measured Stokes parameters. Spatial maps of the degree of polarization (DOP), ψ (azimuth), and χ (ellipticity angle) for (i) 15° and (j) 45° lattices obtained from pixel‐resolved quarter‐wave plate measurements.

## 3 Conclusions

In summary, silica‐coated Fe_3_O_4_ nanorods are used as building blocks to develop twisted liquid‐crystal superlattices and responsive photonic lattices for reconfigurable polarization modulation and moiré polarization optics. Our results have shown that unpolarized light became perpendicularly polarized after interacting with the unidirectional aligned nanorods, in which the performance is dependent on nanorods’ size, concentration, and light wavelength. Twist bilayer liquid crystals along a twist angle generates moiré superstructures in optical polarization space, where systematic rotation of the first layer results in reproducible angular modulation of polarization ellipses, azimuth, and ellipticity, which trace coherent trajectories on the Poincaré sphere. This stacking assembly method provides an effective and flexible way to modulate the rotational and inversion symmetries of the moiré superstructures, leading to highly reconfigurable control over light polarization by a simple structure twist. Lithographically patterned, UV‐cured films encode square and circular liquid crystal patterns with spatially arranged Stokes parameters, enabling pixel‐level reconstruction of the degree of polarization, azimuth, and ellipticity across individual polarization domains. Collectively, these findings show that stacking assembly methods and the emerging moiré photonic phases provide a multifunctional approach to thin, reconfigurable polarizers and flat polarization elements, suggesting potential applications in dynamic photonic modulation and polarization‐encoded anticounterfeiting devices.

## 4 Methods

### 4.1 Preparation and Assembly of Fe_3_O_4_@SiO_2_ Magnetic Nanorods

In total, 10.8 g of FeCl_3_·6H_2_O was dissolved in 400 mL of deionized water and heated to 87°C in an oven for 18 h. After that, FeOOH nanorods precipitated at the bottom, and the supernatant was discarded. Deionized water was used to wash the precipitation three times for 5 min at 11 000 rpm, before dispersing it in 40 mL of DI water. Using PAA, FeOOH nanorods were first modified to form uniform SiO_2_ coatings. The typical method involved mixing 1 mL of a 42 mg/mL PAA (∼2100 MW) solution with 100 mL of DI water, then adding 3 mL of FeOOH aqueous dispersion. After 24 h, we recovered FeOOH nanorods by centrifugation and washed it three times with DI water at 11 000 rpm for 5 min. For the silica coating, 1.5 mL of FeOOH nanorods dispersion was mixed with 1.5 mL of DI water, then added to 20 mL of ethanol, followed by 1 mL of ammonium solution (28%). TEOS (250 µL) was added for the SiO_2_ shell. The mixture was magnetically stirred overnight. FeOOH@SiO_2_ was centrifuged out at 11 000 rpm for 5 min and washed three times with ethanol and water. FeOOH@SiO_2_ was reduced to magnetic nanorods in TTEG at 260°C. In total, 40 mL of DEG was heated to 260°C under nitrogen, to which 250 µL of FeOOH@SiO_2_ aqueous solution was injected. The reduction was kept for 8 h under nitrogen protection. The final product was washed with ethanol and water three times and dispersed in 25 mL of water.

### 4.2 Preparation of the Liquid Crystal Films

For the moiré film preparation, 250 mg of acrylamide (AM), 14 mg of *N*,*N*'‐methylenebisacrylamide (BIS), and 6 µL of 2‐hydroxy‐2‐methylpropiophenone were dissolved in 1 mL of EG. AM is the monomer of the polymer [43], while BIS and 2‐hydroxy‐2‐methylpropiophenone are the crosslinking agent and photoinitiator, respectively. Approximately 1 mL of Fe_3_O_4_@SiO_2_ nanorod colloidal dispersion was centrifuged at 9000 rpm for 3 min, followed by the removal of supernatant. To fully disperse the colloidal nanoparticles, 500 µL of the precursor solution was added and sonicated for 30 s. To prepare solid films, the magnetic nanorod solution was sandwiched between two glass slides, separated by a ∼1 mm spacer [44, 45]. On top of the glass slides was a photomask and a magnetic field was applied. As a result of UV exposure, the orientation of the nanorod was fixed within the film's plane along a specific direction. The glass slide was rotated, flipped, and then again exposed to UV light in a magnetic field. Our result was a thin film with polarization patterns showing different transmittances to polarized light. The films were kept in ethylene glycol (EG) for further characterization.

To produce the complex moiré patterns, the twist (torsion) angle between the top and bottom films was controlled using a manual high precision rotation mount with a graduated scale readable to 1° increments and a vernier resolution of 0.08°. Prior to each measurement, the mount was calibrated against a reference angle set using a fixed alignment pin at the 0° position, confirmed optically by verifying total intensity of transmitted light between the two films co‐aligned in the same direction. This zero‐point calibration was repeated before each data set to correct for any drift introduced by handling or remounting of the sample.

We note that manual rotation introduces higher angular uncertainty than a motorized stage, as the final angle depends on operator set positioning against the graduated scale; a motorized rotation mount would reduce both this operator dependent variability and any systematic backlash error and is recommended for future measurements requiring finer angular resolution. To quantify the achievable accuracy of the manual setup used here, we performed a rotation angle dependent intensity measurement by repeatedly setting and rotating the mount from 0° to 180°. The intensity at each angle was normalized to account for variations in absolute signal range, and the mean normalized intensity was calculated alongside the standard deviation across measurements, which was used as the uncertainty at each angle. The experimental data was then compared to the theoretical cos^2^θ dependence, and the interpolation of the normalized intensity was used to estimate the true rotation angle from the measured intensities (see Figure S17).

### 4.3 Optical Characterization

Mueller matrix polarimetry was used to obtain spatially resolved maps of the polarization properties of the samples. The full Mueller matrix was measured at each pixel using a rotating wave plate configuration, and the resulting matrices were analyzed using standard decomposition methods to extract retardance parameters. From the decomposed matrices, total retardance, linear retardance, circular retardance, DOP, and diattenuation were calculated, where linear retardance corresponds to phase differences between orthogonal linear polarization states and circular retardance is associated with optical activity. Spatial maps of these quantities were then constructed to visualize variations in anisotropy and chiroptical response across the sample.

More specifically, a complete set of 36 polarization‐resolved intensity images was acquired for all combinations of six incident and analyzed polarization states using a polarization state generator (PSG) and a polarization state analyzer (PSA) [46]. From these measurements, each element of the Mueller matrix was constructed through linear combinations (additions and subtractions) of four appropriately selected intensity images. Following Mueller matrix construction, key polarization properties including diattenuation, polarizance, retardance, depolarization, and the angle of polarization were extracted. We applied the Lu–Chipman [47] decomposition to isolate the total retardance component, which quantifies the phase shift imparted by the sample on the orthogonal components of the electromagnetic field. The resulting total retardance matrix was further decomposed into linear retardance (*δ*) and circular retardance (*Ψ*) contributions. The measured retardance was subsequently converted to birefringence using

(1) $$\Delta n=\frac{\lambda \delta }{2\pi t}$$

where *λ* is the illumination wavelength and *t* is the sample thickness.

### 4.4 Calculation of Order Parameter *S*

The dichroic order parameter *S* was determined using polarized UV–vis absorption spectroscopy. Absorbance was measured with the incident light polarized parallel (*A*_∥_) and perpendicular (*A*_⊥_) to the nanorod alignment direction, and the dichroic ratio was defined as

(2) $$R=\frac{A_{∥}}{A_{⊥}}$$

The order parameter was then calculated using

(3) $$S=\frac{R-1}{R+2}$$

In this framework, *S* = 1 corresponds to perfect alignment and *S* = 0 indicates random orientation.

### 4.5 Bra‐ket Notation of the Optical Properties of Twisted Bilayer Liquid Crystals

The linear polarization effect and the polarization state of the twisted bilayers shown in this work can be described using bra‐ket notation. Generally, when light is incident on birefringent materials, it splits into two rays with orthogonal polarization along slightly different optical pathways. Also, for dichroic materials, they absorb light of specific polarization states, converting non‐polarized light into polarized light. With the polarization nature of transmitted light, the intensity can be modulated by two cross‐polarizers. Consider that the light is incident along the *y*‐axis and non‐polarized. The nanorods in the first film (closest to the light source) are magnetically aligned vertically along *z*‐axis. Based on our experiments, the non‐polarized light will become polarized with the direction perpendicular to nanorod alignment. When a film is rotated at an angle of θ relative to *z*‐axis, it defines a new polarization direction in the *x*–*z* plane perpendicular to the nanorods’ alignment. The transmitted polarization state is then

(4) $$\left|\theta \rangle =\;\cos\;\theta \right|x\rangle +\;\sin\theta |z\rangle$$

and the corresponding projector (operator for the first film) is

(5) $$P\left(\theta \right)=\left|\theta \rangle \langle \theta \right|$$

For example, the first film in our experiments contains nanorods with fixed alignment along the *z* axis. The polarization state of light after the first film will be |*x*〉 and the projector at θ = 0 is

(6) $$P^{1}=\left|0\rangle \langle 0\right|=|x\rangle \langle x|$$

In our measurement, the magnetic nanorods in the second film are aligned with at an angle of θ relative to *z*‐axis. We changed the orientation angle (θ) of the second film to control the twist angle of the TBLCs. Specifically, in spherical coordinates, the azimuthal angle (ψ), also known as the orientation, is the polarization direction of the light, while the ellipticity (χ) determines the shape of the polarization ellipse. In this scenario, the polarization azimuthal angle is a parameter in both the Poincaré sphere and the polarization ellipse, denoted as |ψ〉 that can be a linear combination of |*x*>,|*y*〉, and |*z*〉 orthogonal eigenvectors. Therefore, for nanorods, the polarization state can be calculated by applying the second polarization operator (*P* ^2^ = |θ〉〈θ|) on the state function |*x*〉:

(7) $$\begin{array}{cc} \left|\psi \right\rangle & =P^{2}\left|x\right\rangle =\left[\left(\cos\;\theta \left|x\rangle +\sin\theta \right|z\rangle \right)\left(\cos\;\theta \langle x\left|+\sin\theta \langle z\right|\right)\right]\left|x\right\rangle \\ & =\cos\theta |\theta \rangle \end{array}$$

So, the polarization direction is defined by the twist angle of the TBLCs, which is consistent with our observation in Figure 3. The transmittance of the light through the TBLCs at a twist angle of *θ* is proportional to the expectation value of the final polarization state of the transmitted light:

(8) $$\begin{array}{cc} \left\langle \psi \right\rangle & =\langle x|P^{2}\left|x\rangle =\left\langle x\left|\cos\theta \right|\theta \right\rangle =\cos\theta \langle x\right|\left(\cos\;\theta \left|x\rangle +\;\sin\theta \right|z\rangle \right)\\ & =cos^{2}\theta \end{array}$$

This provides a convenient way to predict the polarization state and transmittance of light after interaction with the TBLCs. The kets |*x*〉,|*y*〉, and |*z*〉 satisfy the orthonormality condition.

The physical meaning of operator, |*z*〉〈*z*|, is a perpendicular transmission axis along the *x*–*z* plane. In this simple model, the transmittance of light will be proportional to cos^2^ *θ* and the polarization direction can be predicted by the twist angle *θ*. To verify this theoretical consideration, we measured the transmittance of the TBLCs by consistently changing the twist angle *θ* from 0° to 180°. As shown in Figure S17, the normalized intensity of the transmitted light shows periodic changes against the twist angle. More specifically, we fit the intensity against the twist angle and conclude that the measured intensity of transmitted light after normalization can be predicted by cos^2^ *θ* function, which is consistent by the theoretical consideration developed based on the Bra‐ket notation.

## Author Contributions

Z.L. proposed this work and the experimental design. J.W. ran the stoke parameter analysis, acquired SEM images, and developed the stoke parameter analysis based on bra‐ket notation. R.H. and Z.F. proposed the group symmetry of moiré lattices. A.S. acquired the TEM images. N.R. and O.J. helped prepare and characterized the nanorod samples. D.M. helped with taking optical pictures and data visualization. J.W. and Z.L. prepared the paper. All authors contributed to the discussion of the results.

## Conflicts of Interest

The authors declare no conflicts of interest.

## Supporting information




**Supporting File**: adma74825‐sup‐0001‐SuppMat.docx.

## Data Availability

The data that support the findings of this study are available from the corresponding author upon reasonable request.
