# Supplementary material for: Moiré Phase Mapping in Twisted Bilayer Liquid Crystals with Reconfigurable Light Polarization States
Source: Adv Mater. 2026 Aug 27;38(62):e74825. doi: 10.1002/adma.74825 (PMC13741115; doi:10.1002/adma.74825)
Supplement: Supplementary file 1 [file ADMA-38-e74825-s001.docx]

**Moiré Phase Mapping in Twisted Bilayer Liquid Crystals with Reconfigurable Light Polarization States**

Jordan A. Wilson^1^ | Rui Huang^1^ | Allen Sun^1^ | Zixian Fang^1^ | Doreen Mwangombe^1^ | Naina Rawat^1^ | Oliver Jacobs^1^ | Zhiwei Li^1^*

^1^Department of Chemistry and Biochemistry, University of Maryland, College Park

Correspondence: [zlinano1@umd.edu](mailto:zlinano1@umd.edu)

**Materials**

Iron chloride hexahydrate (FeCl₃·6H₂O), polyacrylic acid (PAA, MW=1800), tetraethyl orthosilicate (C₈H₂₀O₄Si, TEOS), Acrylamide (AM), N,N’-Methylenebisacrylamide (BIS), 2-Hydroxy-2-methylpropiophenone and tetraethylene glycol (TTEG) were purchased from Sigma-Aldrich. Ammonium hydroxide (NH₃·H₂O) was purchased from VWR Chemicals. Ethylene glycol (EG) was from Fisher Scientific. Ethanol was purchased from PHARMCO. All chemicals are used directly without further purification.

**Characterization**

SEM images were acquired using Hitachi SU-70 FEG SEM and TEM images were acquired using JEM 2100 LaB6 TEM. Optical microscopy images were acquired on a Nikon Ti2-E inverted optical microscope. Transmission and adsorption spectra were measured using an Ocean Optics USB4000 spectrometer.

**Figure S1. (a,b)** TEM images of **(a)** FeOOH nanorods and **(b)** Fe₃O₄@SiO₂ nanorods.

**Figure S2.** The orientational order parameter S is determined using polarized UV–Vis absorption spectroscopy by **(a)** measuring absorbance parallel (A_∥_) and perpendicular (A_⊥_) to the nanorod alignment direction to obtain the dichroic ratio R=A_∥_/A_⊥_. **(b)** The dichroic ratio is then used to calculate S.

**Figure S3. (a)** Comparison between experimental and theoretical normalized intensity profiles used to determine the true twist angle θ and correct for rotational misalignment. **(b)** Actual versus estimated rotation angle obtained from interpolation of the normalized intensity.

**Figure S4.**  (a) Schematic illustration of unpolarized light transmitted through a linear polarizer and subsequently through a magnetically aligned film. (b) Transmission spectra as a function of rotation angle for the magnetically aligned film under a linear polarizer. (c) Optical images of the vertically aligned nanorod film under non-polarized light illumination (top), under horizontally polarized light illumination (center), and under vertically polarized light illumination (bottom).

**Figure S5.** Transmittance spectra of liquid crystal films made of nanorods with different sizes: 381 nm × 128 nm for left panels, 410nm x 140nm for middle panels, and 700 nm × 154 nm for right panels, measured under (a) a linear polarized and (b) non-polarized light.

**Figure S6.** Retardance spatial maps of **(a)** 381 nm x 128 nm **(b)** 410 nm x 140 nm and **(c)** 700 nm x 154 nm Fe₃O₄@SiO₂ nanorods.

**Figure S7. (a,b)** Retardance spatial maps of varying Fe₃O₄@SiO₂ nanoparticle concentrations **(a)** 0.82 mg mL^−1^, **(b)** 1.64 mg mL^−1^, **(c)** 2.46 mg mL^−1^**, (d)** 4.92 mg mL^−1^, and **(e)** 6.56 mg mL^−1^

**Table S1.** Birefringence values were calculated from the measured retardance for samples of varying Fe₃O₄@SiO₂ nanorods aspect ratio.

**Table S2.** Birefringence values were calculated from the measured retardance for samples of varying Fe₃O₄@SiO₂ nanorod concentrations.

**Table S3.** Experimentally determined polarizance and diattenuation parameters obtained from Mueller matrix decomposition for samples with varying Fe₃O₄@SiO₂ nanorods aspect ratio.

**Table S4.** Experimentally determined polarizance and diattenuation parameters obtained from Mueller matrix decomposition for samples with varying Fe₃O₄@SiO₂ nanorod concentrations.

**Figure S8.** Dependence of degree of polarization (DOP) of liquid crystal films on wavelength, made of nanorods with dimensions of (a) 381 nm × 128 nm, (b) 410nm x 140nm, and (c) 700 nm × 154 nm.

**Figure S9. (a, b)** Polarization ellipse measurements of nanorods (410 nm × 140 nm) at varying Fe_3_O_4_ nanoparticle concentrations: (a) 1.64 mg/mL and (b) 4.92 mg/mL.

**Figure S10.** Ellipticity (χ) as a function of the twisting angle for the (a) A–A and B–B configurations and (b) B–A and A–B configurations.

**Figure S11.** (a) Demonstration of reading a polarization-encoded pattern using a magnetically aligned thin film placed over a patterned substrate. (b) The underlying domains are not visible under unpolarized illumination alone. Upon stacking, the top film functions as a polarizer, enabling contrast between domains that varies with rotation angle. The polarization-encoded pattern is revealed at rotation angles of (c) 0°, (d) 45°, and (e) 90°. Scale bars: 2 mm.

**Figure S12.** Grayscale optical images of 15° stacked moiré lattices with relative twist angles ranging from 0–80° (top) and 100–180° (bottom), highlighting the evolution of moiré domain morphology with rotation angle.

**Figure S13.** Grayscale optical images of 30° stacked moiré lattices with the quarter-wave plate rotation angle changing from 0–180°, highlighting the evolution of moiré domain morphology with rotation angle. Scale bars: 2 mm.

**Figure S14.** Grayscale optical images of 15° stacked moiré lattices with the quarter-wave plate rotation angle changing from 0–180°, highlighting the evolution of moiré domain morphology with rotation angle. Scale bars: 2 mm.

**Figure S15**. Microscopy images of domain boundaries between regions of differing nanorod orientation: **(a)** vertical, **(b)** narrow transition region, and **(c)** horizontal. The boundary exhibits a sharp transition with a width of ~ 4 μm. Local orientation analysis confirms the narrow interface and high alignment continuity across domains.

**Figure S16.** (a) Spatial maps of the degree of polarization (DOP), ψ (azimuth) and χ (ellipticity angle) for twisted lattices at 30^o^ obtained from pixel-resolved intensity measurements during quarter-wave plate rotation (0°–180°). (b) Four domains in the 30° twisted lattice and (c) their normalized intensity (I_θ_-I_min_)/(I_max_-I_min_) versus quarter-wave plate rotation angle θ. (d) The Poincaré sphere representation of the Stokes vectors for four domains in the 30° lattice. (e) Reconstructed polarization ellipses from the measured Stokes parameters. Scale bars: 2 mm.

**Figure S17.** (a) Poincaré sphere representation of the Stokes vectors for four domains in the 45° lattice. (b) Reconstructed polarization ellipses from the measured Stokes parameters.
